# Genetically Modified *Lactococcus lactis* for Delivery of Human Interleukin-10 to Dendritic Cells

**DOI:** 10.1155/2012/639291

**Published:** 2011-07-27

**Authors:** Inge L. Huibregtse, Sebatian A. Zaat, Martien L. Kapsenberg, Maria A. Sartori da Silva, Maikel P. Peppelenbosch, Sander J. H. van Deventer, Henri Braat

**Affiliations:** ^1^Department of Gastroenterology and Hepatology, Academic Medical Center, 1105 AZ Amsterdam, The Netherlands; ^2^Department of Medical Microbiology, Academic Medical Center,1105 AZ Amsterdam, The Netherlands; ^3^Department of Cell Biology, Histology, and Dermatology, Academic Medical Center, 1105 AZ Amsterdam, The Netherlands; ^4^Department of Gastroenterology and Hepatology, Erasmus Medical Center, P.O. Box 2040, 3000 CA, Rotterdam, The Netherlands; ^5^Department of Gastroenterology and Hepatology, Leiden University Medical Center, 2333 ZA Leiden, The Netherlands

## Abstract

Interleukin-10 (IL-10) plays an indispensable role in mucosal tolerance by programming dendritic cells (DCs) to induce suppressor Th-cells. We have tested the modulating effect of *L. lactis* secreting human IL-10 (*L*.  *lactis* ^*IL*-10^) on DC function in vitro. Monocyte-derived DC incubated with *L*.  *lactis* ^*IL*-10^ induced effector Th-cells that markedly suppressed the proliferation of allogenic Th-cells as compared to *L. lactis*. This suppressive effect was only seen when DC showed increased CD83 and CD86 expression. Furthermore, enhanced production of IL-10 was measured in both *L*.  *lactis* ^*IL*-10^-derived DC and Th-cells compared to *L. lactis*-derived DC and Th-cells. Neutralizing IL-10 during DC-Th-cell interaction and coculturing *L*.  *lactis* ^*IL*-10^-derived suppressor Th-cells with allogenic Th-cells in a transwell system prevented the induction of suppressor Th-cells. Only 130 pg/mL of bacterial-derived IL-10 and 40 times more exogenously added recombinant human IL-10 were needed during DC priming for the generation of suppressor Th-cells. The spatially restricted delivery of IL-10 by food-grade bacteria is a promising strategy to induce suppressor Th-cells *in vivo* and to treat inflammatory diseases.

## 1. Introduction

The gastrointestinal mucosal immune system is continuously balancing between tolerance towards nonpathogenic bacteria and active immunity toward pathogenic bacteria [1]. Animal models have clearly shown that IL-10 is an important regulator in this context as IL-10 knock-out mice develop chronic intestinal inflammation and IL-10 treatment is effective in several models of experimental colitis [2–4]. Unfortunately, several clinical attempts to treat inflammatory bowel disease using IL-10 have failed probably due to low mucosal availability and proinflammatory effects of high-dose systemic IL-10 administration [5–8]. The production of human IL-10 by *Lactococcus lactis* (*L. lactis^IL^* ^-*10*^) could circumvent these problems. Indeed, *L. lactis^IL^* ^-*10 *^ is effective in the prevention and treatment of colitis in animals, and a phase-I clinical trial with *L. lactis^IL^* ^-*10 *^ in Crohn's disease patients suggested clinical benefit [9]. Dendritic cells (DC) are regulators of the adaptive immune system controlling both peripheral tolerance and immune activation [10]. In the absence of pathogenic microorganisms, DC are tuned by microenvironmental factors to induce and maintain tolerance in the intestinal tract. This homeostatic condition is critically controlled by cytokines including IL-10 [11]. Culturing immature DC with IL-10 results in Th-cell responses which suppress the proliferation of allogenic Th-cells in a contact-dependent manner which is not dependent on IL-10 production by (suppressor) Th-cells [12]. We hypothesized that *L. lactis^IL^* ^-*10 *^ is able to modulate immature DC to become regulatory DC which in turn induce suppressor T cells.

## 2. Materials and Methods

### 2.1. Bacterial Strains

For the generation *of Lactococcus lactis* MG1363 IL-10 (*L. lactis^IL^* ^-*10*^) and *Lactococcus lactis* MG1363 (*L. lactis*), the plasmid pOTHY12 was used (Lothar Steidler, ActoGeniX, Belgium). The plasmid contains a 1 Kb region including the constitutive *thyA* promoter (P*thyA*) from L. lactis MG1363, the usp45 secretion leader, hIL-10, and a 1 Kb region downstream of *thyA *gene. This leads to a functional coupling of Pt*hyA* to usp45-hIL-10, expression of the precursor, correct N-terminal processing of the precursor, and secretion of mature hIL-10. In optimized *in vitro* growth, *L. lactis* MG1363 pOTHY12 will produce in its culture supernatant approximately 1 *μ*g hIL-10 per 2 × 10^9^ bacteria. For the generation of the negative control (*L. lactis*), *L. lactis* MG1363 was transformed with an empty plasmid. Both *L. lactis^IL^* ^-*10 *^ and *L. lactis* were grown overnight at 37°C (Elbanton incubator) in M17 broth (Difco) supplemented with 0.5% glucose and 50 *μ*g/mL erythromycin (Abbott, Saint-Rémy-sur-Avre, France). Bacteria were diluted 1 : 50 in IMDM 5% FCS, grown for three hours at 37°C, and harvested at exponential growth phase (approximately 1 × 10^7^ cfu/mL).

### 2.2. Generation and Maturation of DC

All cultures were performed in Iscove's modified Dulbecco's medium (IMDM) with 1% FCS (HyClone, Logan, UT) and erythromycin (50 mg/mL, Abbott). Peripheral blood of healthy volunteers was used to generate immature DC as earlier described [14]. Maturation was achieved at day 6 by adding 1 × 10^5^ cfu bacteria with or without maturation factors (MF), recombinant human (rh)-IL-1**β** (5 ng/mL; Boehringer Mannheim, Germany), rh-TNF-*α* (25 ng/mL; PBH, Hannover, Germany), and LPS (Sigma). After 4 hours, 50 mL of supernatant was harvested for measurement of bacterial IL-10 (ELISA; CLB, Amsterdam, The Netherlands) and gentamycin (86 *μ*g/mL, Sigma) was added to kill all bacteria. On day eight, mature DCs were harvested and washed, and cultures of DC on M17 agars confirmed that bacterial killing was complete. Mature DCs were used for flow cytometry to assess maturation by CD83 and CD86 expression or stimulated with one of the following stimuli: IFN-*γ* (gift from Dr. P van der Meide; U-Cy tech, Utrecht, The Netherlands) and/or CD40L-transfected J558 plasmacytoma cells (gift from Dr. P. Lane, Birmingham Medical School, Birmingham, UK). After 48 hours, supernatants were used for cytokine detection using ELISA for IL12p70 (R&D Systems, detection limit 31.2 pg/mL) and IL-10 (CLB, detection limit 31.2 pg/mL).

### 2.3. T-Cell Instruction by Mature DC

Mature DC (5 × 10^3^ cells/200 *μ*L) were incubated with 2.5 × 10^4^ purified CD4^+^CD45RA^+^CD45RO^−^ naïve T cells (>90% as assessed by flow cytometry) from PBMC using MACS separation system (Miltenyi Biotec, Germany). When indicated rat-antihuman IL-10 neutralizing IgG_1_ (1 : 1000, BD Pharmingen, San Jose, Calif, USA) antibodies were added, purified rat IgG_1_ (BD Pharmingen) was used as isotype control. Th-cells were further expanded and stimulated with soluble mouse antihuman-CD3 and mouse antihuman-CD28 (both CLB). After 48 hours, supernatants were used for cytokine detection using ELISA for IFN-*γ* (R&D Systems, detection limit 31.2 pg/mL) and IL-10 (CLB). Moreover, expanded Th-cells were used in a Th-cell coculture model to assess suppressor function of DC-derived Th-cells [15]. In short, expanded (effector) Th-cells were cultured with CD4^+^ (autologous) T cells and T-cell proliferation was measured using cell-cycle tracking dyes; cell division results in decreased fluorescence intensity of separate cells. The fluorescence intensity of allogenic T cells, after culture with effector T cells derived from MF-matured DC, was taken as 100% (reference condition).

### 2.4. Statistics

For comparison of cytokine production, a heteroscedastic Student *t*-test was performed, while for comparison of percentages, a *X* ^2^-test for comparing percentages was performed. When multiple groups were present a Kruskall-Wallis One-Way Analysis of Variance was performed (SPSS, version 11.01, Chicago, Ill, USA). Statistical significance was defined as a *P* < 0.05, confidence interval 95%.

## 3. Results

### 3.1. L. lactis^(IL-10) ^ Matures DC to Promote the Development of Suppressor T Cells

**Figure 1:**
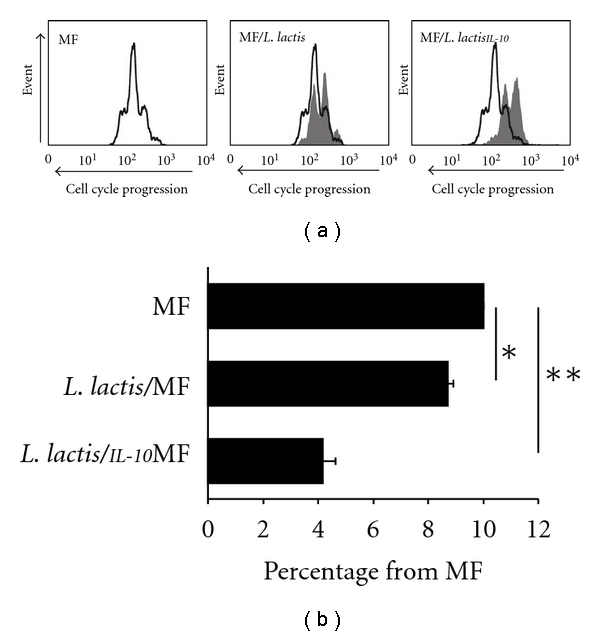
(a) Immature DC were matured with no bacteria, 1 × 10^5^ cfu of viable *L. lactis^IL^* ^-*10 *^or *L. lactis* in the presence of MF. Mature DC were subsequently cultured with naïve Th-cells to induce effector Th-cells which were harvested, washed, and stained with 3 × 10^−5^ M PKH-26, a red cell-cycle tracking dye. Then, 2.5 × 10^4^ effector Th-cells were preactivated overnight with anti-CD3 (1 : 5000) and anti-CD28 (0.5 mg/mL) in round-bottom 96-well plates. The following day allogenic (CD4^+^) Th-cells were labeled with CFSE, a green cell-cycle tracking dye. After 5 days, the cellular content of CFSE in the allogenic Th-cells was analyzed by flow cytometry [13]. Depicted is the cell cycle progression of allogenic Th-cells stimulated in the presence of MF derived effector Th-cells (white histograms, reference condition) and of allogenic Th-cells cultured in the presence of MF/*L. lactis^IL^* ^-*10 *^ or MF/*L. lactis* derived effector Th-cells (gray histograms). (b) Depicted is the mean inhibition of fluorescence intensity of allogenic Th-cells from five separate experiments for the indicated conditions. The proliferation of allogenic Th-cells cultured in the presence of effector Th-cells derived from MF-matured DC which is regarded as 100% (reference condition). Mean percentages with standard error of mean are given; *indicates *P* < 0.05 and **indicates *P* < 0.01 using the *χ* ^2^-test for comparing percentages with the reference condition (MF).

DCs were matured in presence of MF or MF with bacteria (*L. lactis* or *L. lactis^IL^* ^-*10*^) and used to differentiate naïve Th-cells into effector Th-cells. Subsequently, the generated effector Th-cells were analyzed for their effect on Th-cell proliferation in a coculture model [15]. The fluorescence intensity of allogenic Th-cells after culture with effector Th-cells derived from MF-matured DC was taken as 100% (reference condition). Effector Th-cells generated by MF/*L. lactis*-DC allowed the proliferation of 67% of all allogenic Th-cells (Figure 1(a)), while effector Th-cells generated by MF/*L. lactis^IL^* ^-*10 *^-DC only allowed proliferation of 42% of all allogenic Th-cells (Figure 1(a)). The mean proliferation from five separate experiments was 72% (*P* < 0.05) and 42% (*P* < 0.01) for *L. lactis* and *L. lactis^IL^* ^-*10*^, respectively, indicating that IL-10 production by *L. lactis* significantly improved the suppression of allogenic Th-cells proliferation by effector Th-cells (Figure 1(b)).

### 3.2. The Induction of Suppressor Th-Cells by L. lactis^*IL*-*10 *^  Matured DC Is Dependent on Full Maturation of DC

**Figure 2:**
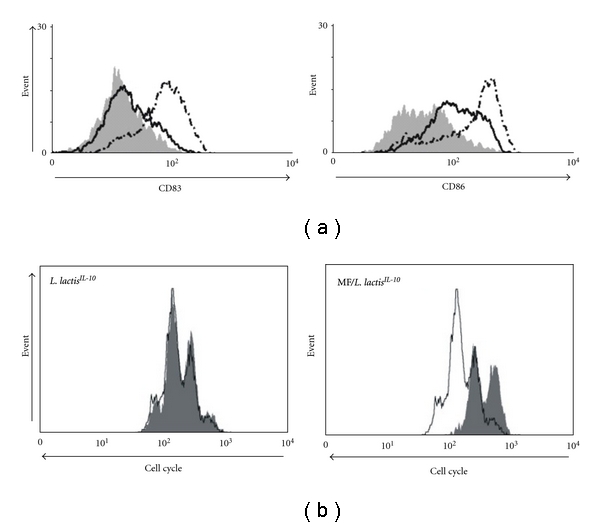
(a) Immature DCs were not matured (grey histogram), matured with 1 × 10^5^ cfu viable *L. lactis^IL^* ^-*10 *^ (continuous line), or matured with MF and 1 × 10^5^ cfu viable *L. lactis^IL^* ^-*10 *^ (dotted line). CD83 (left panel) and CD86 (right panel) expression was assessed after 48 hours by flow cytometry. (b) Immature DCs were primed with MF or 1 × 10^5^ cfu viable *L. lactis^IL^* ^-*10 *^ with and without MF. Depicted is the cell cycle progression of allogenic Th-cells co-cultured with effector Th-cells derived from MF-matured DC is regarded as 100% (white histograms, reference condition) and the cell cycle progression of allogenic Th-cells cocultured with effector Th-cells derived from *L. lactis^IL^* ^-*10 *^ (left panel) or *L. lactis^IL^* ^-*10 *^/MF-(right panel) matured DC (gray histograms).

DC matured with *L. lactis^IL^* ^-*10 *^ in the absence of MF showed a minimal increase in the expression of CD83 and CD86 as expressed by mean fluorescence intensity. When MFs were added during maturation with *L. lactis^IL^* ^-*10 *^ a strong upregulation of CD83 and CD86 was observed as can be seen in the histograms (Figure 2(a)). Compared to the reference condition, DC matured in the absence of MF but in the presence of *L. lactis^IL^* ^-*10 *^-induced effector Th-cells that were not able to significantly suppress naive T-cell proliferation. On the contrary when MF was added together with *L. lactis^IL^* ^-*10 *^ during DC maturation, these DCs were able to induce effector Th-cells that efficiently reduced the proliferation of naïve T cells. Hence, lower expression of CD83 and CD86 in *L. lactis^IL^* ^-*10 *^ matured DC was associated with a marked reduction in their ability to induce effector Th-cells with suppressive effects on allogenic Th-cell proliferation (Figure 2(b)).

### 3.3. L. lactis^*IL*-*10 *^ Matures DC to Produce IL-10 and Induces IL-10 Producing Th-Cells

**Figure 3:**
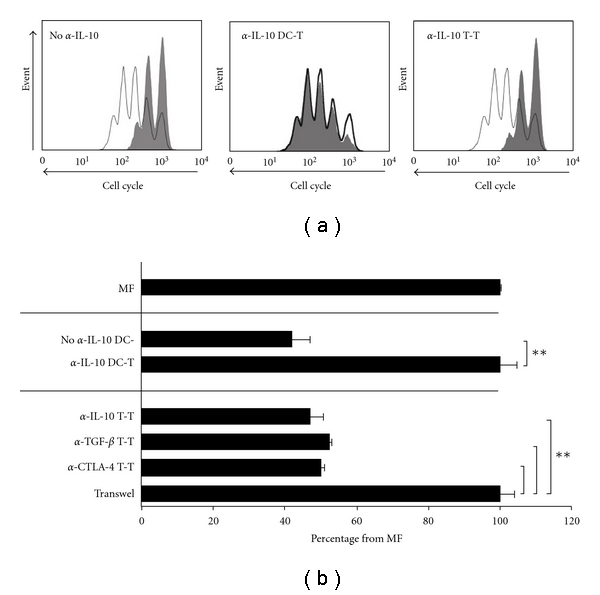
(a) Immature DCs were not matured, matured with MF or with 1 × 10^5^ cfu of viable *L. lactis*, *L. lactis^IL^* ^-*10 *^, *L. lactis^IL^* ^-*10*^/*α*-IL-10, or *L. lactis^IL^* ^-*10 *^/rat IgG_1_ in the presence of MF. Mature DCs were harvested after 48 hours, washed, and restimulated (2 × 10^4^ cells in 200 *μ*L) for 48 hours with CD40L transfected irradiated hybridoma cells to assess IL-10 production or in combination with IFN-*γ* to asses IL-12p70 production. Mean values with standard error of mean are given; * indicates *P* < 0.05 and **indicates *P* < 0.01 using heteroscedastic Student *t*-test. (b) Immature DCs were not matured, matured with MF or with 1 × 10^5^ CFU of viable *L. lactis*, *L. lactis^IL^* ^-*10*^, *L. lactis^IL^* ^-*10 *^/*α*-IL-10 or *L. lactis^IL^* ^-*10 *^/rat IgG_1_ in the presence of MF. Mature DCs were subsequently cultured with naïve Th-cells for 5 days, these DC derived Th-cells were washed and restimulated with CD3/CD28 (100.000 cell/200 *μ*L, final concentration 1 : 1000) to assess IL-10 and IFN-*γ* production. Mean values with standard error of mean are given; **indicates *P* < 0.01 using heteroscedastic Student *t*-test.

*L. lactis^IL^* ^-*10 *^ and *L. lactis*, in combination with MF, were used to mature DC. After 4 hours, bacteria were killed with antibiotics, and after 48 hours, mature DCs were restimulated with CD40L or CD40L/IFN-*γ* to assess IL-10, IL-12p70, and IL-6 production, respectively. DC produced 3200 pg/mL and 1600 pg/mL IL-10 after maturation with *L. lactis^IL^* ^-*10 *^ and *L. lactis,* respectively, (*P* < 0.05, compared to *L. lactis*, Figure 3(a)). The production of IL-12p70 (Figure 3(a)) and IL-6 (data not shown) was not significantly different between the groups. Furthermore, IL-10 and IFN-*γ* production by effector Th-cells, induced by the different DC groups, was measured. Maturation of DC with *L. lactis^IL^* ^-*10 *^ or *L. lactis* in combination with MF resulted in the induction of effector Th-cells that produced 2400 pg/mL and 700 pg/mL IL-10, respectively, (*P* < 0.01, compared to *L. lactis*, Figure 3(b)). The production of IFN-*γ* was not significantly different between the groups (Figure 3(b)). Finally, the addition of neutralizing IL-10 antibodies during maturation of immature DC with *L. lactis^IL^* ^-*10 *^ significantly reversed the production of IL-10 by mature DC and effector Th-cells (*P* < 0.01, compared to isotype control, Figures 3(a) and 3(b)).

### 3.4. IL-10 Is Important for the Induction But Not for the Function of Suppressor Th-Cells

**Figure 4:**
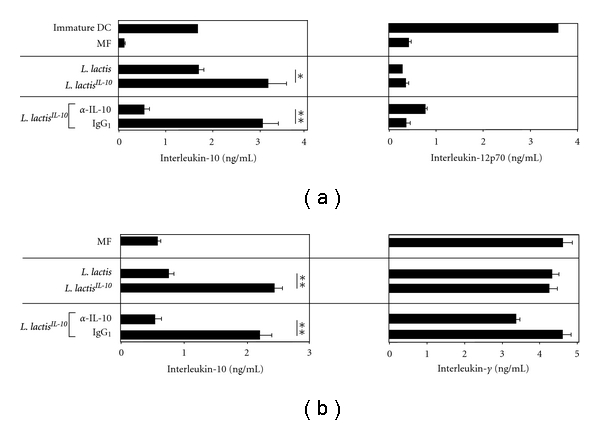
(a) Depicted is the cell cycle progression of allogenic Th-cells cocultured with MF-derived effector Th-cells (white histograms, reference condition) and of allogenic Th-cells cocultured with effector Th-cells when no  *α*-IL-10 was added (no *α*-IL-10), *α*-IL-10 during DC-naïve T-cell (*α*-IL-10 DC-T) coculture or *α*-IL-10 during effector T-cell and allogenic T-cell (*α*-IL-10 T-T) coculture (gray histograms). (b) Bar graphs represent the mean inhibition of fluorescence intensity of allogenic Th-cells for the indicated conditions in triplicate. The proliferation of allogenic Th-cells cocultured with effector Th-cells induced by MF-matured DC is regarded as 100% (reference condition). For transwell experiments, 2 × 10^6^ PKH-labeled test cells were put in the upper compartment of a 24-well plate transwell system. Mean percentages with standard error of mean are given; **indicates *P* < 0.01 using the *χ* ^2^-test for comparing percentages with the reference condition (*α*-IL-10 DC-T and transwell, resp.).

Neutralizing IL-10 during coculture of *L. lactis^IL^* ^-*10 *^-matured DC and naïve Th-cells completely abrogated the suppressive effect of effector Th-cells on allogenic Th-cell proliferation (Figure 4(a)). However, neutralizing IL-10 during coculture of *L. lactis^IL^* ^-*10 *^-derived suppressor Th-cells and allogenic Th-cells did not affect the suppressive effect on allogenic Th-cell proliferation (*P* < 0.01, compared to MF, Figure 4(b)). Also, neutralizing TGF-*β* or CTLA-4 costimulation did not affect the suppressive effect of *L. lactis^IL^* ^-*10 *^-derived suppressor Th-cells on allogenic Th-cells proliferation (*P* < 0.01, compared to MF, Figure 4(b)). Finally, the suppression of *L. lactis^IL^* ^-*10 *^-derived suppressor Th-cells on allogenic Th-cell proliferation was completely abrogated by physical separation of suppressor and allogenic Th-cells (Figure 4(b)).

### 3.5. Efficient Delivery of Bacterial-Derived IL-10 to DC

**Figure 5:**
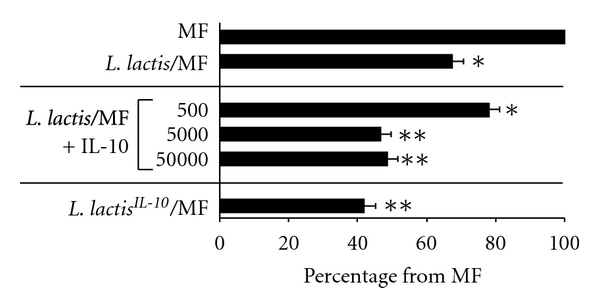
Immature DC primed with MF/*L. lactis* and increasing amounts of recombinant human IL-10 (0, 0.5, 5 and 50 ng/mL). Bar graphs represent the mean inhibition of fluorescence intensity of allogenic Th-cells for the indicated conditions in duplo. The proliferation of allogenic Th-cells cocultured with effector Th-cells derived from MF-matured DC is regarded as 100% (reference condition). Mean percentages with standard error of mean are given; *indicates *P* < 0.05 and **indicates *P* < 0.01 using the *χ* ^2^-test for comparing percentages with the reference condition (MF).

An amount of 10^5^ cfu of *L. lactis^IL^* ^-*10 *^ produced 85 pg/mL of IL-10 in the absence of DC (data not shown). After 4 hours, supernatants from DC cultured with viable *L. lactis^IL^* ^-*10 *^ and MF contained 130 pg/mL (data not shown). Increasing amount of recombinant human IL-10 (500 pg/mL, 5000 pg/mL, and 50.000 pg/mL) during DC cultures with viable *L. lactis* and MF increased the suppressive effect of DC-derived effector Th-cells on allogenic Th-cell proliferation to 67.5% (*P* < 0.05, compared to MF), 46.5% (*P* < 0.01, compared to MF), and 48.5% (*P* < 0.01, compared to MF), respectively, (Figure 5).

## 4. Discussion

The effective ability of DC to initiate different types of Th-cell responses makes this cell type an important target for innovative strategies requiring either polarized immunity (i.e., in vaccination or cancer) or tolerance (i.e., in chronic inflammatory, autoimmune, or allergic diseases) [16]. The immunoregulatory cytokine IL-10 plays a central role in the induction and maintenance of mucosal tolerance through the induction of regulatory DC [1]. However, IL-10 treatment in humans with colitis is disappointing due to low mucosal availability and proinflammatory effects at higher doses of IL-10 [7]. We hypothesized that *L. lactis^IL^* ^-*10 *^ is able to modulate DC to become regulatory DC which in turn induce suppressor T cells. To this aim, we cultured viable *L. lactis* and *L. lactis^IL^* ^-*10 *^ with DC and assessed the effect of DC-derived effector Th-cells on allogenic Th-cell proliferation in the absence of DC and bacteria. We observed that *L. lactis* can imprint a regulatory DC phenotype which is even more pronounced by secretion of IL-10. The final suppressive effect on Th-cell proliferation is probably in part due to the intrinsic immunomodulatory effect of *L. lactis* and in part due to the presence of IL-10 during DC maturation. *L. lactis* has much homology with several probiotic organisms that are known to induce regulatory immune responses [15, 17, 18]. We had to culture *L. lactis^IL^* ^-*10 *^ (temporarily) alive to allow IL-10 production and secretion. Hence, secreted bacterial factors could also have modulated the maturation process, phenotype, and suppressor function of DC. The immunomodulatory effect of IL-10 during priming of DC in our model is in accordance with the findings of other groups [19, 20]. Interestingly, low levels of IL-10 produced by *L. lactis^IL^* ^-*10 *^ during DC maturation are sufficient to induce regulatory DC. *L. lactis^IL^* ^-*10 *^ produced around 130 pg/mL IL-10 before antibiotics were added, and we needed up to 5000 pg/mL of rh-IL-10 to produce comparable suppressive effects. Thus, approximately 40 times more IL-10 during DC priming was needed to induce suppressor Th-cells, suggesting that spatially restricted bacterial IL10 is more efficient than soluble IL-10 for the induction of regulatory DC. It would be interesting to establish if this phenomenon is also occurring *in vivo* and if other factors like bacterial translocation across the epithelium and physical contact with DC in the lamina propria and/or mesenteric lymph node are necessary for inducing these suppressor immune responses [21]. 

The induction of regulatory DC by *L. lactis^IL^* ^-*10 *^ was characterized by full maturation, high production of IL-10, and low production of IL-12p70 and IL-6. Moreover, IL-10 is required for the induction of suppressor Th-cells by DC but not for the suppressive function of suppressor Th-cells during coculture with allogenic Th-cells. The function of IL-10 production by suppressor Th-cells cannot be deduced from our experiments, but it might be important for the persistence of regulatory immune responses through feedback on newly recruited immature DC in the periphery *in vivo* [22]. Unfortunately, we were not able to identify the driving factor behind the suppressor effect of Th-cell induced by *L. lactis^IL^* ^-*10 *^ during T-T cell interaction. Both IL-10 and TGF-**β** are not involved in the suppressive effect of effector Th-cells on naïve T-cell proliferation. The transwell experiment suggests that contact-dependent factors are important, but CTLA-4 was ruled out by using a blocking antibody. Hence, other contact-dependent factors like ICAM-3 and GITR might be important in these effector Th-cells induced by *L. lactis^IL^* ^-*10 *^ [15, 23]. It is an attractive idea to suggest that foxp3 might play an important role in the induction of suppressor Th-cells by *L. lactis^IL^* ^-*10 *^-matured DC, but we have not yet been able to prove such a relationship [24]. 

In conclusion, our data indicate that the observed anti-inflammatory effect of *L. lactis^IL^* ^-*10 *^ is mediated through modulation of DC function. The utilization of *L. lactis* for delivery of proteins has several advantages including increased mucosal availability, ease of administration, lower therapeutic doses, and cost effectiveness. This strategy might be applicable in the treatment of inflammatory and infectious diseases [13, 25, 26].
